# Circulating Interferon-*λ*3, Responsiveness to HBV Vaccination, and HBV/HCV Infections in Haemodialysis Patients

**DOI:** 10.1155/2017/3713025

**Published:** 2017-10-31

**Authors:** Alicja E. Grzegorzewska, Monika K. Świderska, Adrianna Mostowska, Paweł P. Jagodziński

**Affiliations:** ^1^Chair and Department of Nephrology, Transplantology and Internal Diseases, Poznan University of Medical Sciences, Poznan, Poland; ^2^Student Nephrology Research Group, Chair and Department of Nephrology, Transplantology and Internal Diseases, Poznan University of Medical Sciences, Poznan, Poland; ^3^Department of Biochemistry and Molecular Biology, Poznan University of Medical Sciences, Poznan, Poland

## Abstract

The IFN-*λ*3 gene* (IFNL3)* plays a role in HCV clearance. We investigated circulating IFN-*λ*3 and* IFNL3* SNPs in haemodialysis patients who differed in their response to HBV vaccination and their HBV/HCV infection status. In 201 patients, plasma IFN-*λ*3 was determined using ELISA.* IFNL3* SNPs (rs12979860, rs8099917) were genotyped using HRM analysis. Differences in IFN-*λ*3 levels were shown between responders and nonresponders to HBV vaccination and between HBsAg-positive patients and those who developed anti-HBs after infection and became HBsAg negative. HBV vaccine responders without HCV resolution revealed lower IFN-*λ*3 than noninfected responders. HBsAg/HCV RNA-positive subjects showed lower IFN-*λ*3 than patients positive only for HCV RNA or subjects who resolved both infections. Circulating IFN-*λ*3 correlated positively with anti-HBs and negatively with positive HCV RNA testing in the adjusted regression analyses. HBV vaccine nonresponders, HBsAg-positive patients, and subjects with replicating HCV composed a group with unfavourable outcomes. Responders to HBV vaccination, subjects who became HBsAg negative, and those who cleared HCV were analysed as having favourable outcomes. The latter showed higher IFN-*λ*3 but did not differ in distribution of* IFNL3* SNPs compared with subjects with unfavourable outcomes. Higher IFN-*λ*3 concentrations are associated with response to HBV vaccination, self-limited HBV infection, and HCV resolution.

## 1. Introduction

The prevalence rates of hepatitis B virus (HBV) and hepatitis C virus (HCV) infections differ worldwide. Subjects positive for HBV surface antigen (HBsAg) were found to compose approximately 0.20% of the Mexican population and 22.38% of South African inhabitants [1]. Approximately 3% of the world population is infected with HCV [2]. The prevalence of infection is higher in patients with altered immunocompetence. Among immunocompromised individuals, there are patients with severe renal functional damage that requires regular dialysis treatment. In Poland, antibodies against HCV (anti-HCV) were shown in 7.9% and HBsAg positivity in 3.0% of haemodialysis (HD) recipients [3], whereas in the general population the respective percentages were 1% [4] and 0.42% [1]. Although HBV vaccination is now a common practice, dialysis patients are less likely to develop antibodies to HBsAg (anti-HBs) after immunization due to impaired immune system function and tend to lose them faster than healthy vaccinated subjects [5, 6]. A uremic milieu with a related alteration of immunocompetence also promotes the maintenance of HBV/HCV replication [7, 8].

Interferon- (IFN-) *λ*s are potent antiviral cytokines. IFN-*λ*3, also referred to as interleukin- (IL-) 28B, was discovered in early 2003 by two independent groups [9, 10]. It comprises type III subset of IFNs. IFN-*λ*s, which are directly produced in response to HCV infection, inhibit HCV replication [11–13] and are associated with spontaneous resolution and successful treatment of HCV infection [14]. IFN-*λ*s also inhibit HBV replication in a differentiated murine hepatocyte cell line [12]. In a nonuremic Han Chinese population, serum IFN-*λ*3 levels were lower in patients with chronic HBV infection than in subjects with self-limited HBV infection or in healthy subjects [15]. Our earlier study on HD patients demonstrated that circulating IFN-*λ*3 correlates with anti-HBs production after HBV vaccination and infection, but HCV-infected subjects were not excluded from the examined groups and were not separately analysed [16].

IFN-*λ*3 is a protein product of the IFN-*λ*3 gene (*IFNL3*) clustered on chromosome 19 (19q13.13 region, contig AC011445.6). Major homozygosity in* IFNL3* rs12979860 is associated with spontaneous HCV clearance [17, 18] and resolution of HCV infection following treatment with pegylated IFN-*α* and ribavirin [18, 19]. A meta-analysis by Tang et al. [20] found no association between* IFNL3* rs12979860 C/T and persistent HBV infection risk.

Our aim was to investigate circulating IFN-*λ*3 and single nucleotide polymorphisms (SNPs) of* IFNL3* in HD patients who differed in their response to HBV vaccination and occurrence of HBV/HCV infections.

## 2. Patients and Methods

### 2.1. Patients and Controls

A group of 201 HD patients was included in a cross-sectional study. A majority of the patients were dialysed in two dialysis facilities; however, due to the small number of subjects infected with HBV and/or HCV at these two centres, enrolment of patients showing nonresponsiveness to HBV vaccination (anti-HBs < 10 IU/L), as well as patients with positive HBV/HCV seromarkers, was also performed at other 18 dialysis centres in the Greater Poland region of Poland. The inclusion criteria were as follows:

1. age over 18 years,
2. established HBV/HCV seromarkers,
3. established status as a responder or nonresponder to HBV vaccination according to the Advisory Committee on Immunization Practices of the US Centers for Disease Control and Prevention [21],
4. stable clinical condition for at least 2 months prior to enrolment.

Corticosteroid therapy and cachectic conditions causing decreases in serum proteins (neoplasms, enteropathies, and liver cirrhosis), as well as antiviral treatment prior to or at the time of enrolment, were exclusion criteria.

All patients were treated with intermittent HD three times a week, with dialysis sessions lasting approximately four hours each, using low-flux HD, high-flux HD, or on-line haemodiafiltration (HDF).

HD patients who were responders to HBV vaccination were tested for anti-HBs titres every 6 months. If their anti-HBs titres decreased below 10 IU/L or were approaching 10 IU/L, booster vaccine doses were administered to keep the anti-HBs titres over 10 IU/L, the level that is considered protective against HBV infection [21].

HD patients who did not respond to HBV vaccination, those who remained HBsAg positive after infection, and those with replicating HCV composed a group with unfavourable outcomes with respect to HBV vaccination and HBV/HCV infections (*n* = 63, 31.3% of the total). HD subjects who were responders to HBV vaccination, those who became HBsAg negative after HBV infection, and those who spontaneously cleared HCV were included in a group with favourable outcomes (*n* = 138, 68.7% of the total).

Healthy subjects recruited among medical workers and their friends (*n* = 28) having anti-HBs ≥ 10 IU/L after HBV vaccination served as controls.

### 2.2. Laboratory Methods

The blood samples for laboratory measurements were collected before the midweek dialysis session during routinely performed periodical blood examinations appropriate for HD patients.

Plasma IFN-*λ*3 concentration was determined with an enzyme-linked immunosorbent assay (ELISA) kit specific for IFN-*λ*3 (Human Interleukin 28B ELISA Kit, Sunred Biological Technology Co., Ltd., Shanghai, China). The ELISA was performed according to the manufacturer's instructions, and the samples were measured in an ELISA plate reader (Infinite F50, Tecan Group Ltd., Männedorf, Switzerland). Absorbance readings were taken at 450 nm, and results for the test samples were extrapolated from standard curves. The sensitivity of the IFN-*λ*3 ELISA was 0.65 ng/L. The intra-assay coefficient of variation (CV) was <10%, and the interassay CV was <12%.

Anti-HBs titres were determined with microparticle enzyme immunoassay (MEIA) technology (ABBOTT, Germany) or the chemiluminescent microparticle immunoassay (CMIA) method (ABBOTT, Ireland). Titres equal to or exceeding 1,000 IU/L were reported as 1,000 IU/l.

Other laboratory parameters were determined using routine methods.

### 2.3. Genotyping

The* IFNL3* SNPs (rs12979860 C>T and rs8099917 T>G) of all HD patients were genotyped using a high-resolution melting (HRM) curve analysis. The analysis was performed on a LightCycler 480 system (Roche Diagnostics, Mannheim, Germany) with 5x HOT FIREPol EvaGreen HRM Mix (Solis BioDyne, Tartu, Estonia) as previously described [16]. For quality control, approximately 10% of the randomly chosen samples were regenotyped using the same genotyping method; the concordance rate was 100%. Genotyping failed for one sample, and that sample was excluded from further statistical analyses.

### 2.4. Statistical Methods

The results are presented as percentage for categorical variables and medians and range (minimum–maximum) as continuous variables were nonnormally distributed as determined by the Shapiro–Wilk test.

The Mann–Whitney *U* test was used to compare continuous variables. The power of tests comparing IFN-*λ*3 concentrations or anti-HBs titres in subgroups is shown. Adjustment for possible confounding variables was performed using logistic regression. In statistical comparisons, anti-HBs titres ≥ 1,000 IU/L were taken as equal to 1,000 IU/L. Spearman's rank-order correlation coefficient was used to show correlations between selected variables. Pearson's chi-squared test or Fisher's exact test was applied for the comparison of dichotomous variables. The Cochran–Armitage trend test was used to show the significance of trends in the distribution of genotype frequencies between studied groups.

Stepwise logistic regression with backward elimination was applied to select significant variables among other possible determinants of a tested phenotype. A receiver operating characteristic (ROC) curve was plotted to show the area under the curve (AUC) as a measure of the accuracy of the model.

Linear regression was used to determine the associations among circulating IFN-*λ*3 concentrations and patient characteristics. Stepwise linear regression with backward elimination was applied to select independent correlates of circulating IFN-*λ*3.

A *P* value less than 0.05 was considered significant, but the Bonferroni correction was applied for evaluation of* IFNL3* associations. All probabilities were two-tailed.

The previously mentioned statistical analyses were performed using Statistica version 12 (Stat Soft, Inc., Tulsa, OK, USA), R software version 3.4.0 [22], and G*∗*Power 3.1.9.2 (Franz Faul, Universitat Kiel, Germany).

Haplotype frequencies were estimated using the software Haploview 4.2 (http://www.broad.mit.edu/mpg/haploview/). Statistical significance was assessed using the 1000-fold permutation test.

### 2.5. Ethical Approval of Research

The study design was approved by the Institutional Review Board of Poznan University of Medical Sciences, Poland. Written informed consent was obtained from all study participants (201 HD patients and 28 controls).

## 3. Results

### 3.1. HBV and HCV Status of HD Patients

Among the HD patients, 102 (50.7%) were uninfected by hepatotropic viruses, 32 (15.9%) had been exposed to HBV, 35 (17.4%) had been infected with HCV, and 32 (15.9%) showed seromarkers of both infections (Table 1). HBsAg positivity was shown in 8 (4.0%) patients, and anti-HCV positivity was demonstrated by 67 (33.3%) subjects; among them, 39 (57.4%) had replicating HCV (median viral load 2.43*E*5 IU/mL, the range of 0.38*E*5 IU/mL–7.71*E*5 IU/mL) and 28 (41.8%) spontaneously resolved their HCV infections (viral load undetectable).

### 3.2. IFN-*λ*3 and Anti-HBs in HD Patients and Healthy Subjects

Circulating IFN-*λ*3 concentrations, anti-HBs titres, prevalence of nonresponders to HBV vaccination, and prevalence of subjects with anti-HBs titre ≥ 1000 IU/L among subgroups of HD patients stratified by HBV/HCV seromarkers and in healthy subjects are shown in Table 1. Statistical comparisons of variables shown in Table 1 are presented in Table 2.

HBV vaccine responders among noninfected HD patients showed higher IFN-*λ*3 than healthy responders. Anti-HBs titres in these groups were not significantly different (group 2 versus group 22 in Tables 1 and 2).

In the HD group, significant differences in circulating IFN-*λ*3 were shown between noninfected responders and nonresponders to HBV vaccination (group 2 versus group 3 in Tables 1 and 2) as well as between HBsAg-positive patients and those who developed anti-HBs after HBV infection and became HBsAg negative (group 5 versus group 6 in Tables 1 and 2). HBV-infected patients who did not develop anti-HBs included not only subjects who persistently showed positive HBsAg but also individuals with isolated antibodies to HBV core antigen (anti-HBc). The latter group showed neither HBsAg nor anti-HBs but presented anti-HBc positivity. This entire group of anti-HBs-negative subjects exposed to HBV infection showed lower IFN-*λ*3 than patients who became anti-HBs positive after HBV infection. Significantly lower IFN-*λ*3 concentrations in the mentioned groups were accompanied by significantly lower anti-HBs titres (groups 5 and 6 versus group 7 in Tables 1 and 2).

Among anti-HCV-positive subjects, circulating IFN-*λ*3 was not significantly different between patients who resolved HCV infection and those who did not, independently of the subgroups being compared. All HCV RNA-negative patients developed higher anti-HBs titres than HCV RNA-positive subjects. The frequencies of responders and nonresponders to HBV vaccination, as well as subjects with anti-HBs titres ≥ 1000 IU/L, were not significantly different between HCV RNA-negative and HCV RNA-positive individuals (group 9 versus group 10 in Tables 1 and 2). Similarly, HCV RNA-negative responders to HBV vaccination showed higher anti-HBs titres than HCV RNA-positive responders (group 11 versus group 12 in Tables 1 and 2). Moreover, HCV RNA-negative responders showed higher anti-HBs than noninfected responders (group 11 versus group 2 in Tables 1 and 2). HCV RNA-positive responders, compared with noninfected responders, showed similar anti-HBs titres but lower IFN-*λ*3 concentrations (group 12 versus group 2 in Tables 1 and 2).

In patients with both infections, HBsAg-positive/HCV RNA-positive subjects showed lower IFN-*λ*3 than patients who were positive only for HCV RNA (group 16 versus group 17 in Tables 1 and 2) or patients who resolved both infections (group 16 versus group 20 in Tables 1 and 2). Anti-HBs titres did not differ significantly between these groups.

The correlation coefficients between IFN-*λ*3 and anti-HBs titres in relevant subgroups of HD patients and healthy subjects exceeded 0.4 (Table 2).

### 3.3. IFN-*λ*3, IFNL3 SNPs, and HD Patient Outcomes

Characteristics of HD patients with favourable/unfavourable outcomes with respect to HBV vaccination and occurrence of HBV/HCV infections are shown in Table 3. Subjects with favourable outcomes showed higher circulating IFN-*λ*3, also after adjustment for age, duration of renal replacement therapy (RRT), low-flux HD, diabetic nephropathy, chronic glomerulonephritis, anuric status (daily urine output ≤ 100 mL), alanine aminotransferase (ALT), and gamma-glutamyltransferase (GGT). In the best multiple logistic regression model assessed using stepwise backward regression, IFN-*λ*3 was the only positive determinant (OR 1.023, 95% CI 1.013–1.033, *P* = 5.3*E* − 6), whereas chronic glomerulonephritis (OR 0.208, 95% CI 0.079–0.544, *P* = 0.001) and ALT (OR 0.951, 95% CI 0.913–0.990, *P* = 0.015) were negative determinants of favourable outcome with respect to HBV vaccination and occurrence of HBV/HCV infections. The AUC for this model equalled 0.863.

Conversion factors to SI units are as follows: for alanine aminotransferase 1 U/L = 0.0167 *µ*kat/L, for alkaline phosphatase 1 U/L = 0.0167 *µ*kat/L, for aspartate aminotransferase 1 U/L = 0.0167 *µ*kat/L, and for gamma-glutamyltransferase 1 U/L = 0.0167 *µ*kat/L

In the entire HD group, there were no significant differences in circulating IFN-*λ*3 with respect to* IFNL3* SNPs (Table 4), and patients with favourable outcomes did not differ in distribution of* IFNL3* SNPs compared with subjects with unfavourable outcomes, although some association was suggested by the *P*_genotype_ (Table 5). It is noteworthy that associations between* IFNL3* SNPs and spontaneous HCV clearance were observed in our study; however, because of the small number of HCV-infected patients, they were not significant after the Bonferroni correction (*P* = 0.018 for CT + TT versus CC rs12979860 C>T, and *P* = 0.012 for GG + GT versus TT rs8099917 T>G).

If low-flux HD patients were analysed separately from those treated with more efficient dialysis techniques (high-flux HD and HDF), associations with both tested phenotypes (IFN-*λ*3 and outcome) yielded better significance (Tables 6 and 7). However, when the Bonferroni correction was applied, significance was no longer detected. Such differences were not shown among patients treated with high-flux HD or those treated with HDF.

*IFNL3* haplotype frequencies were not different between patients showing favourable and unfavourable outcomes (all *P* values > 0.27). There were also no differences when haplotypes were compared between outcomes separately among patients treated with low-flux HD (all *P* values > 0.21) or high-flux HD and HDF (all *P* values > 0.21).

In univariate analyses, circulating IFN-*λ*3 correlated positively with anti-HBs titre and negatively with duration of RRT, anuric status, HCV RNA positivity, and HBsAg positivity (Table 8). Variables associated with IFN-*λ*3 concentrations in univariate analyses were included in the multiple linear regression analysis. After stepwise backward selection, the best model showed anti-HBs titre, HCV RNA positivity, and HBsAg positivity as independent explanatory correlates of circulating IFN-*λ*3 (multiple *R* squared for the model: 0.293, adjusted *R* squared for the model: 0.282, *P* value: 9.4*E* − 15).

To show the values of the tested variables with respect to circulating IFN-*λ*3 concentration, we compared patient characteristics after stratification of all patients according to tertiles of IFN-*λ*3 level (Table 9). The results of this approach showed that patients with IFN-*λ*3 levels in the lower tertile demonstrated higher prevalence of positive tests for HBsAg and anti-HCV, a lower anti-HBs titre, and a lower frequency of favourable outcomes than patients in the upper tertile of IFN-*λ*3 levels.

Conversion factors to SI units are as follows: for alanine aminotransferase 1 U/L = 0.0167 *µ*kat/L, for alkaline phosphatase 1 U/L = 0.0167 *µ*kat/L, for aspartate aminotransferase 1 U/L = 0.0167 *µ*kat/L, and for gamma-glutamyltransferase 1 U/L = 0.0167 *µ*kat/L.

## 4. Discussion

Comparison of responders to HBV vaccination among HD patients free of HBV/HCV infections and healthy subjects, all being responders to HBV vaccination, showed that IFN-*λ*3 is upregulated in HD patients. In uremic rats, 2665 of 10,153 genes were differentially expressed, with 47% up- and 53% downregulated [23]. IFN-*λ*3 may be upregulated in a uremic milieu; however, a lower degradation rate is also a possibility, as with many other biologically active proteins [24–27]. In our study, anuric status (daily urine output ≤ 100 mL) seemed to negatively influence IFN-*λ*3 concentrations, but this correlation was not statistically significant after adjustment for the variables that were significant in univariate analyses. Moreover, circulating IFN-*λ*3 levels did not correlate with urine output in HD patients with preserved residual renal function and anuric patients did not differ in IFN-*λ*3 concentrations from those with daily diuresis over 100 mL (data not shown). This evidence suggests no relationship between IFN-*λ*3 levels and urine output in HD patients.

Circulating IFN-*λ*3 concentrations are shown to be associated with response to HBV vaccination [16] as well as with resolution of HBV [15] and HCV [14] infections. We investigated associations between plasma IFN-*λ*3 and anti-HBs titres in HD patients not infected with HBV/HCV, infected with either HBV or HCV alone, and exposed to both HBV and HCV.

In HBV/HCV-noninfected responders to HBV vaccination among HD patients, there was a significant positive correlation between IFN-*λ*3 and anti-HBs titres, as in healthy responders. It is not clear whether there is a direct causal association between IFN-*λ*3 and anti-HBs formation after vaccination. Indoleamine 2,3-dioxygenase (IDO) might be involved. IDO gene activity is directly induced by IFN-*λ*3 in a dose-dependent manner [28]. Therefore, more efficient anti-HBs production in the presence of higher circulating IFN-*λ*3 levels might occur due to IFN-*λ*3-induced upregulation of IDO expression. A mouse study showing that IDO inhibition at the time of vaccination with HBsAg decreased anti-HBs titre is in accordance with this concept [29]. Additionally, increased IDO activity seems to skew T helper (Th) cell polarization towards a Th2 pathway, which primarily results in antibody formation [30, 31]. The addition of IFN-*λ*3 to immunizations in mice caused a greater than 2-fold increase in the titres of IgG2a antigen-specific antibodies produced after Th1 pathway stimulation. An increased release of the Th1-associated cytokine IFN-*γ* was also observed [32]. In HD patients, a deficit in IFN-*γ* was noted despite the increased blood levels of Th1 cytokines involved in IFN-*γ* production, such as IL-18 [33]. Moreover, when used as an adjuvant during vaccination against a lethal influenza virus in mice, IFN-*λ*3 induced 100% protection from mortality associated with this viral infection [32]. However, these results were not confirmed in rhesus macaques when the generation of IgG2a antigen-specific antibodies was examined following the addition of IFN-*λ*3 to the immunization strategy [34]. It remains unknown how IFN-*λ*3 is stimulated by immunizations with HBsAg.

A positive correlation of IFN-*λ*3 with anti-HBs titre was also demonstrated in HD patients who were able to generate anti-HBs in response to HBV infection. We postulate that HBV might be a trigger for IFN-*λ*3 production and that rising IFN-*λ*3 concentrations promote anti-HBs development. A study by Sato et al. [35] demonstrated that type III IFNs, including IFN-*λ*3, are induced in human hepatocytes during HBV infection. Retinoic acid-inducible gene 1 (RIG-1) senses the HBV genotypes A, B, and C for the induction of type III IFNs through its recognition of the 5′-*ε* region of HBV-derived pregenomic RNA. In primary human hepatocytes, significant HBV-related induction was observed for IFN-*λ*1, IFN-*λ*2, and IFN-*λ*3 mRNAs. Moreover, after chimeric mice were intravenously infected with HBV genotype C derived from chronic hepatitis B patients, the expression of IFN-*λ*1, IFN-*λ*2, and IFN-*λ*3 mRNAs in the liver tissue was found to be increased at 4 or 5 weeks after infection [35]. Additionally, IDO elevations were observed in acute hepatitis B patients with self-limited HBV infection [36]. After HBV infection, HBsAg clearance is usually associated with the development of anti-HBs, although the anti-HBs response alone cannot account for HBsAg clearance [37, 38]. In a nonuremic Han Chinese population, serum IFN-*λ*3 levels were lower in patients with chronic HBV infection than in subjects with self-limited HBV infection or healthy subjects. A gene expression microarray analysis showed enhanced IFN-*λ*3 expression in patients with a low HBV viral load [15]. In our previous study [16], HBV-infected patients who had severe renal damage requiring HD treatment and were not able to generate anti-HBs included subjects with chronic hepatitis B and subjects with isolated anti-HBc positivity. These combined groups showed lower IFN-*λ*3 than subjects who developed anti-HBs after infection. This finding was also relevant in the current study. Moreover, IFN-*λ*3 was a significant independent predictor of HBsAg clearance in the HD group. Therefore, the current study demonstrates that, in HD patients, higher circulating IFN-*λ*3 levels may be associated with self-limited HBV infection.

An endogenous IFN response is activated during HCV infection; however, IFN induction, IFN signalling, and transcription of IFN-stimulated gene mRNA are not efficient in hepatocytes from chronic hepatitis C patients [39]. Circulating IFN-*λ*3 levels may be associated with the outcome of HCV infection. In a study by Shi et al. [18], patients with persistent HCV infection (median viral load 11.2*E*5 IU/mL) showed lower circulating IFN-*λ*3 than patients who cleared their infections or healthy controls. Subjects who spontaneously cleared HCV revealed similar circulating IFN-*λ*3 levels to a healthy group [40]. In a study by Aoki et al. [41], chronic hepatitis C patients demonstrated substantially higher IFN-*λ*3 levels than healthy volunteers. After treatment with pegylated IFN-*α* and ribavirin, the increased IFN-*λ*3 levels returned to normal values in patients who cleared their HCV, but treatment effects were not related to baseline circulating IFN-*λ*3 levels [41]. In these two aforementioned studies, renal function was not mentioned and was probably normal. Similarly, there were no data on HBV vaccination in these studied groups. In our study, there were no significant differences in circulating IFN-*λ*3 between patients with spontaneous HCV resolution and HCV-infected patients when all patients, responsive or unresponsive to HBV vaccination, were compared within HCV-exposed subgroups. However, although not significantly different (perhaps as a result of small sample sizes), differences in IFN-*λ*3 suggested lower IFN-*λ*3 in all HCV RNA-positive patients and in HCV RNA-positive responders compared with HCV RNA-negative subjects. Moreover, responders to HBV vaccination with persistent HCV infection showed lower IFN-*λ*3 than infection-free responders, whereas responders who resolved HCV infection showed similar IFN-*λ*3 levels to infection-free responders. The latter group may represent a “control” status among HD subjects, given that all patients were free from HBV/HCV infections and were able to respond to HBV vaccination, which is a predictor of longer survival in HD population [42]. Additionally, IFN-*λ*3 was a significant independent negative explanatory variable for HCV RNA positivity in the entire HD group, which argues for an association between higher IFN-*λ*3 and spontaneous HCV resolution. Therefore, our results are more consistent with those of Shi et al. [18]. However, patients examined by Aoki et al. [41] probably showed more severe HCV disease course (all had a diagnosis of chronic hepatitis C, the group showed a high mean viral load of 2.0*E*6 IU/mL, and all patients required antiviral therapy), whereas our patients were diagnosed as long-term HCV carriers with lower viral load and were not qualified for IFN-based therapy. This patient-related difference may be important for IFN-*λ*3 levels. In accordance with this discrepancy are data showing that* IFNL3* alleles favourable for spontaneous HCV resolution may be associated with increased inflammation and higher fibrosis scores in HCV-infected subjects [40].

Among population with normal renal function, responsiveness to HBV vaccination is lower in subjects infected with HCV, whereas individuals who cleared HCV show higher vaccine effectiveness than subjects not exposed to HCV [43]. In the studied HD patients, responsiveness to HBV vaccination was similar in noninfected and HCV-infected patients, but HBV vaccine responders who did not resolve their HCV infection showed significantly lower anti-HBs titres than did responders with spontaneous HCV resolution. The latter group showed even higher anti-HBs titres than noninfected responders. Although HCV infection status divided HD patients into those with spontaneous HCV resolution and those showing persistent HCV infection, a significant positive correlation between IFN-*λ*3 and anti-HBs being generated in response to HBV vaccination was observed in both subgroups, independently of HCV outcome.

In the previous studies, homozygosity for the major allele (i.e., the CC genotype) in* IFNL3* rs12979860 was attributed to spontaneous HCV clearance [17, 18, 40] and resolution of HCV infection following treatment with pegylated IFN-*α* and ribavirin [18, 19]. A study by Yu et al. [44] showed that* IFNL3* rs8099917 affects spontaneous HCV clearance among Taiwanese HD patients. HD subjects bearing the rs8099917 allele G or the rs12979860 allele T had a lower chance of spontaneous HCV clearance [45]. In HD patients, comparison of responders and nonresponders to HBV vaccination revealed no significant differences in the* IFNL3* genotype distribution; in HBV-infected patients, differences in the distribution of* IFNL3* variants were also not significant between anti-HBs-negative and anti-HBs-positive patients [45]. In this study,* IFNL3* SNPs were significantly associated with neither HD patient outcomes nor circulating IFN-*λ*3, but homozygosity for the major alleles of rs8099917 or rs12979860 was associated with HCV clearance, as shown in many previous studies [17, 18, 40, 44, 45].

In noninfected human hepatocytes, higher expression levels of* IFNL3* were found in cells with the TT genotype of* IFNL3* rs12979860 than in cells with the CC genotype [46]. Pretreatment liver biopsy specimens from chronic HCV patients revealed higher expression levels of IFN-*λ*1 and IFN-*λ*3 in patients with the* IFNL3* rs12979860 TT genotype than in those with the CC genotype. This higher expression of IFN-*λ*1 and IFN-*λ*3 was accompanied by higher expression of IFN-*λ* receptor and antiviral IFN-stimulated genes [46]. These results seem to indicate that the TT genotype might be associated also with higher circulating IFN-*λ*3, as suggested in low-flux HD patients in the current study. However, a combined population of subjects with persistent HCV, individuals who spontaneously cleared HCV, and healthy controls exhibited higher IFN-*λ*3 serum levels in subjects showing major homozygosity of rs12979860 [18]. Interestingly, subjects with persistent HCV (an unfavourable outcome by our criteria) composed 63.0% of all subjects in this group.

In this study, we did not show association of* IFNL3* rs8099917 with circulating IFN-*λ*3 in the entire group of HD patients, which is in agreement with the results obtained by Aoki et al. [41] from Japanese patients with chronic hepatitis C. Lower intrahepatic expression of* IFNL3* mRNA in HCV-infected liver recipients and donors was demonstrated in subjects harbouring a variant allele of rs8099917 [47]. Expression levels of* IFNL3* in peripheral blood mononuclear cells of HCV-infected subjects were also lower in patients who carried the variant alleles of rs8099917 [47, 48] and were comparable with those in the liver [47]. In HD patients treated using low-flux dialysers, lower circulating IFN-*λ*3 was also shown in bearers of a variant allele of rs8099917 [16]. On the other hand, subjects living in the Riyadh area of Saudi Arabia showed higher serum IFN-*λ*3 levels if they were homozygous for a variant allele of rs8099917, whereas the major allele of rs8099917 was found to be correlated with reduced IFN-*λ*3 serum levels [49].

We would like to stress that higher circulating IFN-*λ*3 levels were independently associated with favourable outcomes for HD patients with respect to responsiveness to HBV vaccination, and therefore with better protection of HD patients against HBV, as well as with respect to self-limited HBV infection and spontaneous HCV clearance, and therefore with avoidance of serious liver complications related to persistent HBV/HCV viremia (hepatic cirrhosis and hepatocellular carcinoma) [50–53]. The association of circulating IFN-*λ*3 with the response status and the strength of the response to HBV vaccination or infection provides arguments for further studies dealing with the subject of why HBV/HBsAg may be a trigger for IFN-*λ*3 and anti-HBs development in some patients, whereas other subjects are not responsive.

## 5. Study Limitations

The main limitation of our study is the small number of patients in each subgroup. Therefore, only very evident differences surfaced as statistically significant. To show a statistically significant difference in IFN-*λ*3 levels between HCV RNA-negative and HCV RNA-positive responders to HBV vaccination with a test power of 80%, we would have needed a 2.1-fold greater number of responders in each group, assuming the same proportion of HCV RNA-positive and HCV RNA-negative responders as in the already-studied HCV-exposed responders. Given the fine division of the patients into many subgroups, the number of patients in the entire sample needs to be much higher than what was available in the current study. Our study is regional; whole-country assessment might be helpful in enrolling more satisfactory subgroups.

Determination of plasma IFN-*λ*3 concentration is not routinely used and at present there are no recommendations of which test should be used for IFN-*λ*3 level quantifications. In our studies ([16], this study), we applied the Chinese ELISA kit. The USA ELISA test for IFN-*λ*3 was used by Li et al. [15] and Shi et al. [18]. Aoki et al. [41] quantified IFN-*λ*3 by chemiluminescence enzyme immunoassay (CLEIA), which in healthy subjects yielded the lowest IFN-*λ*3 levels compared with those presented by the other mentioned authors ([15, 16, 18], this study). The Chinese ELISA kit provided lower IFN-*λ*3 levels than those obtained by the USA ELISA kit. However, studies using the USA ELISA kit also did not show comparable results in healthy Chinese controls [15, 18]. Therefore, IFN-*λ*3 levels seem to depend on the method and even on the test used for IFN-*λ*3 determination. We did not perform studies to establish the sensitivity and specificity of the used assay; however, further laboratory studies could specify which IFN-*λ*3 concentrations describe true IFN-*λ*3 levels.

## 6. Conclusions

1. Circulating IFN-*λ*3 is upregulated in HD responders to HBV vaccination compared with healthy responders.
2. In HD patients, responders to HBV vaccination show higher circulating IFN-*λ*3 levels than nonresponders.
3. HD patients with self-limited HBV infection show higher IFN-*λ*3 plasma concentrations than persistently HBsAg-positive subjects.
4. In HD patients, spontaneous HCV clearance is associated with higher IFN-*λ*3 levels; however, stimulation of the immune system by HBV vaccination for maintenance of protective anti-HBs titres may contribute to increases in IFN-*λ*3 as anti-HBs production is associated with higher circulating IFN-*λ*3.
5. In HD patients, IFN-*λ*3 is crucial for protection against HBV and for self-limitation of hepatotropic infections.

## Figures and Tables

**Table 1:** Circulating IFN-*λ*3, anti-HBs titre, prevalence of nonresponders to HBV vaccination, and subjects with anti-HBs titre ≥ 1000 IU/L among subgroups of HD patients stratified by HBV/HCV seromarkers and healthy subjects.

Number	HBV/HCV seromarkers	*N*	Male gender, *n* (% of all)	Age, years	IFN-*λ*3, ng/L	Anti-HBs titre, IU/L	Nonresponders to HBV vaccination, *n* (% of all)	Subjects with anti-HBs titre ≥ 1000 IU/L, *n* (% of all)
*1*	*HD patients uninfected with HBV/HCV*	*102*	*54 (52.9)*	*67.5 (29–88.2)*	*90.6 (15.9–232.7)*	*188 (0–1474)*	*17 (16.7)*	*20 (19.6)*
2	Responders to HBV vaccination	85	45 (52.9)	67.1 (29–88.2)	120 (36–232.7)	262 (10–1474)	0 (0)	20 (23.5)
3	Nonresponders to HBV vaccination	17	9 (52.9)	68 (54.8–85.1)	43 (15.9–77.4)	2 (0–9.8)	17 (100)	0 (0)
*4*	*HD patients infected with HBV (anti-HBc positive)*	*32*	*16 (50)*	*71.8 (27.4–91)*	*107.1 (10–215.4)*	70.2 (0–1000)	N/A	7 (21.9)
5	HBsAg positive	4	2 (50)	52.8 (27.4–57.9)	39.1 (10–83.4)	0 (0–3)	N/A	0 (0)
6	HBsAg negative/anti-HBs positive	25	12 (48)	72.3 (43–91)	125 (35–215.4)	187 (12.4–1000)	N/A	7 (28)
7	Isolated anti-HBc	3	2 (66.7)	78.7 (56.5–90)	70.9 (49–125)	0.5 (0–2)	N/A	0 (0)
*8*	*HD patients infected with HCV (anti-HCV positive)*	*35*	*16 (45.7)*	*62.2 (31.3–88.1)*	*71.2 (4.9–400)*	*354.5 (0–1000)*	*6 (17.1)*	*11 (31.4)*
9	All HCV RNA negative	14	6 (42.9)	66.3 (35.1 –88.1)	91.8 (12.3–400)	905 (2–1000)	2 (14.3)	7 (50.0)
10	All HCV RNA positive	21	10 (47.6)	58.2 (31.3 –77.8)	58.5 (4.9–275)	222 (0–1000)	4 (19.0)	4 (19.0)
11	HCV RNA-negative responders to HBV vaccination	12	5 (41.7)	65.6 (35.1 –88.1)	133.2 (14.8–400)	1000 (81–1000) 344.7 (30–1000)	0 (0)	7 (58.3)
12	HCV RNA-positive responders to HBV vaccination	17	9 (52.9)	53.5 (31.3–71.8)	74 (4.9–275)		0 (0)	4 (23.5)
13	HCV RNA-negative nonresponders to HBV vaccination	2	1 (50)	77 (70.1–83.9)	17.4 (12.3–22.4)	3.5 (2–5)	2 (100)	0 (0)
14	HCV RNA-positive nonresponders to HBV vaccination	4	1 (25)	62.1 (60.1–69.4)	43.1 (31–109)	0.7 (0–3.5)	4 (100)	0 (0)
*15*	*HD patients infected with HBV and HCV*	*32*	*20 (62.5)*	*59.1 (24.4–80.3)*	*55 (9–300)*	*195.5 (0–1000)*	*N/A*	*10 (31.3)*
16	HBsAg positive/HCV RNA positive	3	1 (33.3)	53.9 (41.2–55.5)	13.3 (9–21.6)	2 (0–564.7)	N/A	0 (0)
17	Anti-HBc positive/anti-HBs positive /HCV RNA positive	13	9 (69.2)	59.8 (35.8–73.9)	57.5 (13.7–203)	137.2 (28–1000)	N/A	5 (38.5)
18	HBsAg positive/anti-HCV positive/HCV RNA negative	1	1 (100)	39.9	106.2	0	N/A	0 (0)
19	Anti-HBc positive/anti-HBs negative/HCV RNA positive	2	2 (100)	61.4 (51.4–71.4)	24 (24–24)	2	N/A	0 (0)
20	Anti-HBc positive/anti-HBs positive/anti-HCV positive/HCV RNA negative	11	5 (45.5)	59.5 (24.4–80.3)	88.5 (16.0–300)	762.3 (9.7–1000)	N/A	5 (45.5)
21	Anti-HBc positive/anti-HBs negative/anti-HCV positive/HCV RNA negative	2	2 (100)	66.6 (65–68.3)	134.4 (40–228.8)	3	N/A	0 (0)
*22*	*Healthy subjects with positive postvaccination anti-HBs*	*28*	*11 (39.3)*	*56.8 (20.7–77.7)*	*62.4 (12.3–280)*	*442 (10–1000)*	*0 (0)*	*8 (28.6)*

Anti-HBc: antibodies against the core antigen of hepatitis B virus, anti-HBs: antibodies against the surface antigen of hepatitis B virus, anti-HCV: antibodies against hepatitis C virus, HBsAg: surface antigen of hepatitis B virus, HBV: hepatitis B virus, HCV: hepatitis C virus, HCV RNA: ribonucleic acid of hepatitis C virus, HD: haemodialysis, and IFN: interferon.

**Table 2:** Statistical analysis of data shown in Table 1 among subgroups 1–21 of HD patients and healthy subjects (group 22).

Compared subgroups	*P* value for				Test power, %		Compared subgroups	Spearman's rank-order correlation coefficient between IFN-*λ*3 and anti-HBs titre	*P* value
	IFN-*λ*3	Anti-HBs titre	Prevalence of nonresponders to HBV vaccination	Prevalence of subjects with anti-HBs titre ≥ 1000 IU/L	IFN-*λ*3	Anti-HBs titre			
2 versus 3	9.9*E* − 8	7.8*E* − 11^a^	N/A	N/A	99.9	99.9	1	0.619	4.3*E* − 12
2 versus 11	0.714	0.005^a^	N/A	0.033^b^	28.8	87.8	4	0.672	2.6*E* − 5
2 versus 12	0.013	0.882^a^	N/A	1.000^b^	54.9	5.2	8	0.610	0.0001
2 versus 22	4.1*E* − 4	0.973^a^	N/A	0.592^c^	91.0	6.8	15	0.549	0.002
5 versus 6	0.010	0.002^a^	N/A	N/A	90.7	68.9	22	0.812	1.6*E* − 7
5 and 7 versus 6	0.008	6.6*E* − 5^a^	N/A	N/A	90.7	87.3	2	0.435	3.1*E* − 5
9 versus 10	0.321	0.033^a^	1.000^b^	0.073^b^	40.9	66.4	6	0.577	0.003
11 versus 12	0.127	0.018^a^	N/A	0.119^b^	48.7	76.8	11	0.617	0.033
11 versus 13	0.055	0.036^a^	N/A	N/A	45.4	98.5	12	0.504	0.039
12 versus 14	0.347	0.003^a^	N/A	N/A	16.0	75.7	11 + 12	0.572	0.001
13 versus 14	0.105	0.247^a^	N/A	N/A	24.5	17.9	17	0.928	1.3*E* − 5
16 versus 17	0.031	0.123^a^	N/A	N/A	58.8	15.3	20	0.458	0.157
16 versus 20	0.020	0.056^a^	N/A	N/A	62.5	45.1	17 + 20	0.682	0.0003

^a^Mann–Whitney test; ^b^Fisher's exact test; ^c^Pearson's chi-squared test. Anti-HBs: antibodies against the surface antigen of hepatitis B virus, HBV: hepatitis B virus, HD: haemodialysis, IFN: interferon, and N/A: not applicable.

**Table 3:** Characteristics of HD patients showing unfavourable or favourable outcomes with respect to HBV vaccination and occurrence of HBV/HCV infections.

Parameter	Unfavourable outcomes *n* = 63 (31% of all)	Favourable outcomes *n* = 138 (69% of all)	*P* value
*Demographic data*			
Male gender, *n*, % of all	35 (55.6)	71 (51.4)	0.589^a^
Age, years	62.0 (27.4–85)	67.3 (24.4–91)	0.003^c^
RRT duration, years	7.2 (0.1–30.2)	4.9 (0.2–23.1)	0.030^c^
*Cause of ESRD*			
Diabetic nephropathy, *n*, % of all	11 (17.5)	46 (33.3)	0.021^a^
Hypertensive nephropathy, *n*, % of all	10 (15.9)	23 (16.7)	0.888^a^
Chronic glomerulonephritis, *n*, % of all	24 (38.1)	19 (13.8)	9.6*E* − 5^a^
Chronic tubulointerstitial nephritis, *n*, % of all	4 (6.3)	14 (10.1)	0.382^a^
*Type of RRT*			
Low-flux HD, *n*, % of all	40 (63.5)	70 (50.7)	0.092^a^
High-flux HD, *n*, % of all	21 (33.3)	64 (46.4)	0.082^a^
HDF, *n*, % of all	2 (3.2)	4 (2.9)	1.000^b^
PD as the first modality of RRT, *n*, % of all	3 (4.8)	4 (2.9)	0.680^b^
*Daily urine output ≤ 100 mL*, *n*, % of all	52 (82.5)	91 (65.9)	0.016^a^
*Laboratory data*			
IFN-*λ*3, ng/L	50.7 (4.9–275)	120 (14.8–400)	1.4*E* − 11^c,d^
HBsAg positivity, *n*, % of all	8 (12.7)	0 (0)	6.7*E* − 5^b^
Anti-HBc positivity, *n*, % of all	23 (36.5)	41 (29.7)	0.337^a^
Anti-HBs titre, IU/L	9.8 (0–1000)	299.3 (0–1474)	4.1*E* − 8^c^
Anti-HCV positivity, *n*, % of all	42 (66.7)	25 (18.1)	1.3*E* − 11^a^
HCV RNA positivity, *n*, % of all	37 (58.7)	0 (0)	<2.2*E* − 16^a^
ALT, IU/L	18 (4–90)	13 (1–57)	0.005^c^
AST, IU/L	16 (6–94)	15 (6–43)	0.084^c^
GGT, IU/L	41 (7–355)	26 (5–513)	0.003^c^
ALP, U/L	113.5 (50.5–803.8)	95.9 (13.5–1353.3)	0.132^c^
C-reactive protein, mg/L	5.1 (0.1–128.4)	6.0 (0–142)	0.192^c^

^a^Pearson's chi-squared test; ^b^Fisher's exact test; ^c^Mann–Whitney *U* test; ^d^the  *P* value after adjustment for age, RRT vintage, low-flux HD, diabetic nephropathy, chronic glomerulonephritis, urine output, ALT, and GGT using logistic regression was 6.7*E* − 6. ALP: alkaline phosphatase, ALT: alanine aminotransferase, anti-HBs: antibodies against the surface antigen of hepatitis B virus, anti-HBc: antibodies against the core antigen of hepatitis B virus, anti-HCV: antibodies against hepatitis C virus, AST: aspartate aminotransferase, ESRD: end-stage renal disease, GGT: gamma-glutamyltransferase, HBsAg: surface antigen of hepatitis B virus, HCV: hepatitis C virus, HD: haemodialysis, HDF: haemodiafiltration, IFN: interferon, *N*: number of patients, PD: peritoneal dialysis, RRT: renal replacement therapy.

**Table 4:** Circulating IFN-*λ*3 in relation to *IFNL3* polymorphic variants in all HD patients.

Tested SNP	Major homozygote *n* (frequency) IFN-*λ*3, ng/L	Heterozygote *n* (frequency) IFN-*λ*3, ng/L	Minor homozygote *n* (frequency) IFN-*λ*3, ng/L	Mode of inheritance	*P* value
*IFNL3* rs8099917	TT	GT	GG	GG + GT versus TT	0.136
	101 (0.51)	89 (0.45)	10 (0.05)	GG versus GT + TT	0.327
	79.9 (10–264)	109 (4.9–400)	66 (13.7–207)	TT versus GG	0.523
*IFNL3* rs12979860	CC	CT	TT	TT + CT versus CC	0.606
	67 (0.34)	83 (0.42)	50 (0.25)	TT versus CT + CC	0.064
	79 (10–264)	86 (4.9–400)	98.6 (9–215.4)	CC versus TT	0.100

**Table 5:** *IFNL3* polymorphic variants in HD patients showing different outcomes with respect to HBV vaccination and HBV/HCV infections.

	Unfavourable outcomes (*n*, frequency) *n* = 62	Favourable outcomes (*n*, frequency) *n* = 138	Odds ratio (95% CI)	*P* value^a^	*P*_trend_^b^	*P*_genotype_^a^
*IFNL3 rs12979860*						
CC	18 (29)	49 (35.5)	*Reference*	—	0.583	0.027
CT	34 (54.8)	49 (35.5)	1.889 (0.943–3.785)	0.071		
TT	10 (16.1)	40 (29.0)	0.681 (0.283–1.639)	0.389		

CT + TT versus CC	44 (71.0)	89 (64.5)	1.346 (0.703–2.578)	0.370		
TT versus CC + CT	10 (16.1)	40 (29.0)	0.471 (0.218–1.018)	0.052		

MAF	(0.44)	(0.47)	0.879 (0.574–1.347)	0.554		

*IFNL3 rs8099917*						
TT	30 (48.4)	71 (51.4)	*Reference*	—	0.754	0.933^c^
GT	29 (46.8)	60 (43.5)	1.144 (0.618–2.117)	0.668		
GG	3 (4.8)	7 (5.1)	1.014 (0.246–4.189)	1.000^c^		

GG + GT versus TT	32 (51.6)	67 (48.6)	1.130 (0.621–2.059)	0.689		
GG versus GT + TT	3 (4.8)	7 (5.1)	0.952 (0.238–3.809)	1.000^c^		

MAF	(0.28)	(0.27)	1.073 (0.669–1.723)	0.769		

MAF: minor allele frequency. ^a^Pearson's chi-squared test; ^b^Cochran–Armitage trend test; ^c^Fisher's exact test.

**Table 6:** Circulating IFN-*λ*3 in relation to *IFNL3* polymorphisms in patients treated with low-flux HD.

Tested SNP	Major homozygote *n* (frequency) IFN-*λ*3, ng/L	Heterozygote *n* (frequency) IFN-*λ*3, ng/L	Minor homozygote *n* (frequency) IFN-*λ*3, ng/L	Mode of inheritance	*P* value
*IFNL3* rs8099917	TT	GT	GG	GG + GT versus TT	0.365
	55 (0.50)	48 (0.44)	7 (0.06)	GG versus GT + TT	0.845
	87.3 (10–264)	109.2 (9–400)	74 (48.5–207)	TT versus GG	1.000
*IFNL3* rs12979860	CC	CT	TT	TT + CT versus CC	0.770
	31 (0.28)	44 (0.40)	35 (0.32)	TT versus CT + CC	0.027^a^
	95.6 (10–264)	80.8 (16–400)	115.1 (9–207)	CC versus TT	0.188

^a^Not significant after the Bonferroni correction (*P* > 0.004).

**Table 7:** *IFNL3* polymorphic variants in HD patients treated with low-flux HD showing different outcomes with respect to HBV vaccination and HBV/HCV infections.

	Unfavourable outcomes (*n*, frequency) *n* = 40	Favourable outcomes (*n*, frequency) *n* = 70	Odds ratio (95% CI)	*P* value^a^	*P*_trend_^b^	*P*_genotype_^a^
*IFNL3 rs12979860*						
CC	10 (25)	21 (30)	*Reference*	—	0.254	0.011^c^
CT	23 (57.5)	21 (30)	2.300 (0.883–5.993)	0.086		
TT	7 (17.5)	28 (40)	0.525 (0.171–1.608)	0.256		

CT + TT versus CC	30 (75)	49 (70)	1.286 (0.534–3.098)	0.575		
TT versus CC + CT	7 (17.5)	28 (40)	0.318 (0.124–0.819)	0.015^c^		

MAF	(0.46)	(0.55)	0.704 (0.406–1.222)	0.212		

*IFNL3 rs8099917*						
TT	20 (50)	35 (50)	*Reference*	—	0.616	0.519^d^
GT	19 (47.5)	29 (41.4)	1.147 (0.516–2.546)	0.737		
GG	1 (2.5)	6 (8.6)	0.292 (0.033–2.599)	0.406^d^		

GG + GT versus TT	20 (50)	35 (50)	1.000 (0.460–2.175)	1.000		
GG versus GT + TT	1 (2.5)	6 (8.6)	0.274 (0.032–2.358)	0.419^d^		

MAF	(0.26)	(0.29)	0.859 (0.464–1.593)	0.630		

MAF: minor allele frequency. ^a^Pearson's chi-squared test; ^b^Cochran–Armitage trend test; ^c^not significant after the Bonferroni correction (*P* > 0.004); ^d^Fisher's exact test *IFN-λ3 correlates*.

**Table 8:** Explanatory and response correlates of the circulating IFN-*λ*3 concentration among demographic, clinical, and laboratory data of the entire group of HD patients (*n* = 201).

Parameter	Unadjusted		Adjusted^b^	
	*β* ^a^ ± SE	*P* value	*β* ^a^ ± SE	*P* value
Male gender	−10 ± 10.4	0.340	0.7 ± 9.0	0.939
Age (per 10 years)	−1.9 ± 3.5	0.581	−3.5 ± 3.2	0.288
RRT vintage (per 1 year)	−1.7 ± 0.8	0.037	−0.2 ± 0.9	0.861
Diabetic nephropathy	1.6 ± 11.6	0.892	−9.4 ± 10.0	0.347
Hypertensive nephropathy	3.1 ± 14.1	0.825	4.5 ± 12.1	0.710
Chronic glomerulonephritis	−6.2 ± 12.7	0.629	9.8 ± 11.8	0.407
Chronic tubulointerstitial nephritis	18 ± 18.3	0.327	8.9 ± 15.6	0.569
LF-HD	7.3 ± 10.5	0.489	11.1 ± 9.0	0.222
PD as the first modality of RRT	−17.2 ± 28.5	0.546	23.6 ± 25.0	0.347
Daily urine output ≤ 100 mL	−24.8 ± 11.4	0.030	−13.1 ± 10.8	0.230
HBsAg positivity	−71.3 ± 26.2	0.007	−41.9 ± 23.8	0.079
Anti-HBc positivity	8.7 ± 11.2	0.439	1.5 ± 10.2	0.880
Anti-HCV positivity	−15 ± 11	0.177	−5.7 ± 13.5	0.675
Anti-HBs titre (per 100 IU/L)	8.9 ± 1.1	3.6*E* − 14	8.4 ± 1.1	6.4*E* − 13
HCV RNA positivity	−36.7 ± 13.2	0.006	−28.5 ± 12.9	0.028
ALT (per 1 IU/L)	−0.8 ± 0.4	0.066	−0.1 ± 0.4	0.780
AST (per 1 IU/L)	−0.4 ± 0.5	0.423	0.2 ± 0.4	0.701
GGT (per 1 IU/L)	−0.03 ± 0.08	0.713	0.06 ± 0.07	0.426
ALP (per 1 IU/L)	−0.03 ± 0.03	0.385	−0.02 ± 0.03	0.438
C-reactive protein (per 1 mg/L)	−0.06 ± 0.30	0.831	−0.005 ± 0.256	0.985

^a^ *β* coefficient and SE values can be interpreted as follows: for a unitary change in an analysed parameter, the circulating IFN-*λ*3 concentration would change by *β* ± SE (ng/L). ^b^Adjusted for RRT vintage, anti-HBs titre, HBsAg positivity, HCV RNA positivity, and urine output, as appropriate. ALP: alkaline phosphatase, ALT: alanine aminotransferase, anti-HBc: antibodies against the core antigen of hepatitis B virus, anti-HBs: antibodies against the surface antigen of hepatitis B virus, anti-HCV: antibodies against hepatitis C virus, AST: aspartate aminotransferase, GGT: gamma-glutamyltransferase, HBsAg: surface antigen of hepatitis B virus, HCV RNA: ribonucleic acid of hepatitis C virus, HD: haemodialysis, IFN: interferon, LF-HD: low-flux haemodialysis, PD: peritoneal dialysis, and RRT: renal replacement therapy.

**Table 9:** Characteristics of HD patients stratified by tertiles of circulating IFN-*λ*3 concentration.

Parameter	Tertiles of circulating IFN-*λ*3 concentration			*P* value	*P* for trend test
	Lower (≤64 ng/L) *N* = 67	Middle (65–123 ng/L) *N* = 68	Upper (≥124 ng/L) *N* = 66		
*Demographic data*					
Male gender, *n*, % of all	39 (58.2)	36 (52.9)	31 (47.0)	0.430^a^	0.194
Age, years	64.9 (27.4–88.1)	66.2 (29.5–88.2)	66.4 (24.4–91)	0.840^b^	
RRT duration, years	5.6 (0.1–29.9)	5.2 (0.2–30.2)	5 (0.3–26.5)	0.722^b^	
*Cause of ESRD*					
Diabetic nephropathy, *n*, % of all	21 (31.3)	16 (23.5)	22 (30.3)	0.418^a^	0.805
Hypertensive nephropathy, *n*, % of all	11 (16.4)	11 (16.2)	11 (16.7)	0.997^a^	0.969
Chronic glomerulonephritis, *n*, % of all	13 (19.4)	18 (26.5)	12 (18.2)	0.448^a^	0.868
Chronic tubulointerstitial nephritis, *n*, % of all	8 (11.9)	2 (2.9)	8 (12.2)	0.102^a^	0.978
*Type of RRT*					
Low-flux HD, *n*, % of all	34 (50.7)	35 (51.5)	41 (62.1)	0.337^a^	0.489
High-flux HD, *n*, % of all	31 (46.3)	29 (42.6)	25 (37.9)	0.617^a^	0.328
HDF, *n*, % of all	2 (3.0)	4 (5.9)	0 (0)	0.169^c^	0.315
PD as the first modality of RRT, *n*, % of all	3 (4.5)	2 (2.9)	2 (3.0)	0.900^c^	0.648
*Daily urine output ≤ 100 mL*, *n*, % of all	50 (74.6)	53 (77.9)	40 (60.6)	0.064^a^	0.075
*Laboratory data*					
HBsAg positivity, *n*, % of all	6 (9.0)	2 (2.9)	0 (0)	0.021^c^	0.008
Anti-HBc positivity, *n*, % of all	24 (35.8)	17 (25)	23 (34.8)	0.328^a^	0.899
Anti-HBs titre, IU/L	26.3 (0–1000)	192 (0–1474)	960.3 (0–1000)	1.9*E* − 13^b^	
Anti-HCV positivity, *n*, % of all	33 (49.3)	16 (23.5)	18 (27.3)	0.003^a^	0.007
HCV RNA positivity, *n*, % of all	20 (60.6)	11 (16.2)	6 (33.3)	0.080^a^	0.096
ALT, IU/L	15 (3–90)	14 (3–52)	12 (1–57)	0.106^b^	
AST, IU/L	15 (6–94)	15 (7–68)	15.5 (6–43)	0.750^b^	
GGT, IU/L	35 (7–213)	29.5 (5–441)	24 (10–513)	0.202^b^	
ALP, U/L	99.8 (54.3–1109.5)	98.5 (41–1299.3)	105.9 (13.5–1353.3)	0.808^b^	
C-reactive protein, mg/L	5.2 (0.1–128.4)	7.3 (0.5–105.5)	5.1 (0–142)	0.187^b^	
*Favourable outcomes*	26 (38.8)	52 (76.5)	60 (90.9)	1.8*E* − 10^a^	8.9*E* − 11

^a^Pearson's chi-squared test; ^b^Kruskal–Wallis rank sum test; ^c^Fisher's exact test. ALP: alkaline phosphatase, ALT: alanine aminotransferase, anti-HBs: antibodies against the surface antigen of hepatitis B virus, anti-HBc: antibodies against the core antigen of hepatitis B virus, anti-HCV: antibodies against hepatitis C virus, AST: aspartate aminotransferase, ESRD: end-stage renal disease, GGT: gamma-glutamyltransferase, HBsAg: surface antigen of hepatitis B virus, HCV: hepatitis C virus, HD: haemodialysis, HDF: haemodiafiltration, IFN: interferon, *N*: number of patients, PD: peritoneal dialysis, and RRT: renal replacement therapy.
